# Characterisation of Soil Bacterial Communities That Exhibit Chemotaxis to Root Exudates from Phosphorus-Limited Plants

**DOI:** 10.3390/microorganisms11122984

**Published:** 2023-12-14

**Authors:** Katherine V. Weigh, Bruna D. Batista, Huong Hoang, Paul G. Dennis

**Affiliations:** School of the Environment, The University of Queensland, Brisbane, QLD 4072, Australiab.durantebatista@uq.edu.au (B.D.B.);

**Keywords:** rhizodeposition, communication, microbiome, ecosystem services, rhizosphere

## Abstract

The ability to sense and direct movement along chemical gradients is known as ‘chemotaxis’ and is a common trait among rhizosphere microorganisms, which are attracted to organic compounds released from plant roots. In response to stress, the compounds released from roots can change and may recruit symbionts that enhance host stress tolerance. Decoding this language of attraction could support the development of microbiome management strategies that would enhance agricultural production and sustainability. In this study, we employ a culture-independent bait-trap chemotaxis assay to capture microbial communities attracted to root exudates from phosphorus (P)-sufficient and P-deficient *Arabidopsis thaliana* Col-0 plants. The captured populations were then enumerated and characterised using flow cytometry and phylogenetic marker gene sequencing, respectively. Exudates attracted significantly more cells than the control but did not differ between P treatments. Relative to exudates from P-sufficient plants, those collected from P-deficient plants attracted a significantly less diverse bacterial community that was dominated by members of the *Paenibacillus*, which is a genus known to include powerful phosphate solubilisers and plant growth promoters. These results suggest that in response to P deficiency, Arabidopsis exudates attract organisms that could help to alleviate nutrient stress.

## 1. Introduction

Phosphorus (P) is essential for plants, contributing significantly to vital processes like energy generation, nucleic acid synthesis, and photosynthesis. After nitrogen, P is the second most limiting nutrient in agriculture [1]. This is because plants generally acquire P as orthophosphate ions from the soil solution, where it is then replenished from the soil’s solid phase or via the decomposition of organic matter, albeit at rates often much slower than plant demand [2]. To reduce P limitation, plants have evolved a variety of biochemical, physical, and ecological strategies that help to optimise its use and enhance its acquisition [1]. These include the production and release of organic acids and phosphatases [3]; altered root growth and morphology [4]; and the formation of symbioses with phosphate-solubilising (PS) soil microbes such as mycorrhizal fungi and PS rhizobacteria [5,6]. Mycorrhizae, for example, have extensive hyphal networks that greatly extend the mined volume of soil, and along with diverse bacteria and other microbes, help mobilise P into solution via the release of protons, organic acids, and phosphatases [5]. Generally, these strategies aim to enhance P solubilisation chemically and enzymatically, to maximise the volume of soil mined for P, and to increase the uptake of P by the plant root. Hence, soils harbour a wealth of microbial taxa that could be used to more sustainably maintain or enhance global food security. To achieve this, novel approaches to ‘engineer’ the composition of root-associated microbial communities must be developed.

The assembly of rhizosphere microbial communities is strongly associated with the quantity and composition of organic carbon-containing compounds released from the roots (rhizodeposits) [7]. Rhizodeposits include a wide range of organic molecules that attract or repel chemotactic soil organisms that are able to sense their presence and direct movement accordingly [8]. This ‘chemical language’ has coevolved to mutually enhance the fitness of plants and microbes to prevailing biotic and abiotic conditions [5]. For example, when under attack, the activation of jasmonic acid-mediated plant defences is associated with changes in the composition of root exudates and rhizosphere bacterial communities [9]. These changes not only confer protection to the plant but also warn nearby plants about a potential threat [10].

Shifts in the composition of rhizodeposits and root microbiomes have also been observed in association with a wide range of abiotic stresses such as salinity [11], drought [12], and nutrient deficiencies [13]. Some of the taxa recruited by stressed plants include bacteria containing 1-aminocyclopropane-1-carboxylate (ACC) deaminase, which suppresses ethylene levels in response to stress, including drought [14,15,16]. Furthermore, in addition to increasing plant nutrient uptake via the release of protons, organic acids, enzymes, and siderophores [17,18,19], many of the taxa associated with the roots of nutrient-deficient plants release auxins (e.g., indole-3-acetic acid) and gibberellins [20] that increase root surface area. Hence, in response to stress, coevolution between plants and soil microorganisms appears to have enabled plants to ‘signal for help’ from their symbionts when experiencing adverse conditions. Decoding this language of attraction, i.e., better understanding the relationships between exudates and the microbial taxa that they attract would greatly support the development of approaches for manipulating root microbiomes and enhancing plant resilience under stress. One way to achieve this is by analysing how chemotactic rhizosphere microorganisms respond to exudates. The way this is achieved, however, typically relies on traditional capillary assays that focus on single-cultured bacterial isolates [21,22]. Hence, due to the poor scalability of this approach, our understanding of soil microbial responses to exudates at the community level is limited, and the relative intensity of these responses is unknown.

In this study, we demonstrate the use of a novel bait-trap assay [23,24] to capture soil microorganisms that exhibit chemotaxis to root exudates collected from control (P-sufficient) and P-deficient *Arabidopsis thaliana* Col-0 plants. The trap consists of a plastic syringe loaded with root exudates, which is then immersed in a soil extract to capture chemotactic microbes. As the trapped organisms respond to the same resource, they may be considered to represent ecological guilds. Flow cytometry and metabarcoding were used to enumerate and characterise the diversity of these guilds, respectively. Hence, this approach facilitates the concomitant detection of diverse taxa and their relative responses to exudates.

## 2. Materials and Methods

### 2.1. Plant Growth Conditions

*Arabidopsis thaliana* Col-0 seeds were surface-sterilised via a 15 h exposure to chlorine gas generated by adding concentrated hydrochloric acid (1 M) to bleach (4% NaClO) up to a final concentration of 6.1% [25]. Seeds were then (i) incubated at 4 °C in the dark for 72 h on full-strength Murashige Skoog (MS) media (1 mM KH_2_PO_4_, 1 mM MgSO_4_, 0.25 mM K_2_SO_4_, 0.25 mM CaCl_2_, 2 mM NH_4_NO_3_, 0.1 mM Na-Fe-EDTA, 50 μM KCl, 30 μM H_3_BO_3_, 5 μM MnSO_4_, 1 μM ZnSO_4_, 1 μM CuSO_4_, 0.7 μM NaMoO_4_, and pH 5.7) with 1% sucrose and 0.7% agar, and (ii) transferred to a 23 °C growth room with a photoperiod of 16 h light (130 μmol m^−2^ s^−1^) and 8 h dark. After eight days, seedlings were transferred to six-well plates (one seedling per well) containing 5 mL of half-strength MS liquid media with 1% (*w*/*v*) sucrose per well. Plates were placed on orbital shakers at 90 rpm in a 23 °C growth room with a photoperiod of 16 h light (130 μmol m^−2^ s^−1^) and 8 h dark. The nutrient solution was replaced every three days, at which point the sterility of axenic growth conditions was verified by plating 10 μL of each of the discarded solutions onto Tryptic Soy Agar (TSA, 1% Agar). Any contaminated plants were discarded.

### 2.2. Collection of Root Exudates from P-Sufficient Control and P-Deficient Plants

After 18 days of growth (bolting stage), the plants were washed with sterile water and transferred to new six-well plates, with each well containing 5 mL of half-strength MS liquid media with KH_2_PO_4_ (P-sufficient control treatment) or without KH_2_PO_4_ (P-deficient treatment). Plates were then returned to the orbital shakers at 90 rpm in a 23 °C growth room with a photoperiod of 16 h light (130 μmol m^−2^ s^−1^) and 8 h dark. After three days of growth under control or P-deficient conditions, the nutrient solutions were replaced with sterile deionised water for a 5 h period [26]. The water containing the root exudates released during that period was collected and passed through 0.22 µm syringe filters (Millipore, Billerica, MA, USA) to remove root and microbial cells. This process was repeated to collect root exudates from all plants on three consecutive days. The wells were refilled with 5 mL of either P-sufficient control or P-deficient nutrient solution between exudate extractions. The sterility of the axenic growth conditions was confirmed by plating 10 μL of each of the discarded nutrient solutions onto Tryptic Soy Agar (TSA, 1% Agar) prior to the addition of water for exudate collection. Any contaminated plants and their exudates were discarded. In the end, root exudates were collected from at least seven non-contaminated technical replicates (i.e., individual plants) per biological replicate for both the P-sufficient control and P-deficient treatments. Hence, seven technical replicates per biological replicate were pooled, each yielding 35 mL exudate extracts that were freeze-dried and stored at −80 °C.

### 2.3. Capture of Soil Microbes That Exhibit Chemotaxis to Root Exudates

Soil microorganisms that exhibited chemotaxis towards root exudates were captured using a novel ‘bait-trap’ assay [23,24], where the exudates were used as the ‘bait’ and syringes containing the bait served as the ‘trap’.

### 2.4. Preparing the Source Community

A soil sample was collected (0–20 cm depth) from a Pineapple farm in Queensland, Australia (27.02° S, 152.92° E). This soil has been described in our previous work [27]. Briefly, the soil is classified as Kandosol [28], which is also known as an Ultisol [29]. The sampled soil had a sandy loam texture, pH of 5.4 (1:5 soil/water), and a total organic carbon content of 1.1%.

Microorganisms were dislodged from soil particles by vortexing 5 g of soil in a 50 mL tube containing sterile 1X PBS for 10 s. The slurry was then passed through a 40 µm sieve to separate large particles resulting in an extract containing soil microorganisms, which was diluted 10X by the addition of sterile 1X PBS. This final extract was used as the ‘source community’ for all subsequent assays.

### 2.5. Preparing the Control and Exudate-Baited Trap Solutions

An aliquot of the ‘source community’ was passed through a 0.22 µm syringe filter to remove microbial cells and soil particles. This filtered soil extract was used as the ‘non-baited’ trap solution for control treatments. In addition, this filtered soil extract was used to resuspend the freeze-dried root exudates, resulting in a final concentration approximately 90 times higher than the originally collected exudate concentration. These ‘exudate-baited’ trap solutions were used for both the P-sufficient control and P-deficient treatments.

### 2.6. Setting the Traps

Sterile 60 mL plastic containers were filled with the source community solution (15 mL per container). Three 1 mL syringes containing 0.2 mL of either P-sufficient or P-deficient plant exudates were fixed into the cap of each container. To perform the chemotaxis assay, the syringe tips were submerged into the source community solution for 30 min. Control assays (trap solution containing only filtered soil extract) were conducted in parallel to verify the influence of soil solution on chemotaxis and random microbial movement into the traps. During the incubation, microbes that exhibited chemotaxis towards the bait compounds migrated into the traps. Each syringe served as one technical replicate. After the incubation, we retrieved the contents of three syringes per treatment and used them for cell enumeration by flow cytometry. Additionally, we pooled the contents of nine syringes to form three biological replicates per treatment, which were used for 16S rRNA gene amplicon sequencing.

### 2.7. Enumeration of Chemotactic Microbial Cells by Flow Cytometry

The contents of the submerged syringes were diluted with a 1:2 ratio using filtered soil extract. Non-baited control samples were kept undiluted. All samples were analysed by an Accuri C6 flow cytometer fitted with an FITC 200 filter and a 488 nm laser to count cells trapped as events per µL.

### 2.8. Identifying the Composition and Relative Abundance of Microbial Populations

#### 2.8.1. DNA Extraction, PCR, and Sequencing

Three replicate samples per treatment were pooled (200 µL final volume) and centrifuged at 10,000 rcf for 15 min. After removing the supernatant, pelleted cells were resuspended in 20 µL microLYSIS (Clent Life Science, Stourbridge, UK) and genomic DNA was extracted according to the manufacturer’s instructions. Library preparation was performed by amplifying the bacterial 16S rRNA gene with primer pair 926F (5′-AAA CTY AAA KGA ATT GRC GG-3′) and 1392wR (5′-ACG GGC GGT GWG TRC-3′) [30]. Forward and reverse primers were modified on the 5′ end to incorporate the Illumina overhang adapter for compatibility with i5 and i7 Nextera XT indices, respectively. PCRs were carried out in 20 μL reactions containing 12.6 μL ultra-pure water, 4.0 μL 5× Phire^®^ buffer, 0.4 μL 10 μM dNTPs, 0.3 μL 10 μM forward primer, 0.3 μL 10μM reverse primer, 0.4 μL Phire^®^ Hot Start II, and 2 μL of template DNA. PCR was performed on a Veriti^®^ 96-well Thermocycler (Applied Biosystems, Australia) with the following conditions: initial denaturation at 98 °C for 45 sec, 35 cycles of 5 s at 98 °C, 5 s at 56 °C, and 6 s at 72 °C, followed by a final extension of 1 min at 72 °C. Amplicon size (∼450bp), and the quality was confirmed by gel electrophoresis using a 1.5% agarose gel.

PCR products were purified using the Agencourt AMPure XP system (Beckman Coulter, Inc., Brea, CA, USA) and subjected to dual indexing using the Nextera XT Index Kit (Illumina) as per the manufacturer’s instructions. Indexed amplicons were then purified as previously described and quantified using Qubit™ dsDNA HS Assay kit (Thermo Fisher Scientific, Waltham, MA, USA). Purified PCR products were pooled at equimolar concentrations and sequenced on an Illumina MiSeq at the University of Queensland’s Institute for Molecular Biosciences using 30% PhiX Control v3 (Illumina, San Diego, CA, USA) and a MiSeq Reagent Kit v3 (600 cycle; Illumina) according to the manufacturer’s instructions.

#### 2.8.2. Bioinformatic Processing

Data were analysed using a modified UPARSE pipeline [31] as described by Forstner et al. [27]. Briefly, USEARCH was used to (1) remove primers and trim residual sequences to 250bp using fastx_truncate; (2) quality filter sequences using fastq_filter (-fastq_maxee = 1); (3) remove duplicate sequences using fastx_uniques; (4) cluster sequences at 97% similarity; and (5) generate an OTU table from the pre-trimmed reads and OTU representative sequences using otutab with default parameters. SILVA SSU (v138) [32] taxonomy was assigned to the cluster representatives using BLASTN (v2.7.1) [33]. The number of reads was then rarefied to 4450 per sample. The diversity of bacterial OTUs (Shannon) in each sample were calculated using QIIME2 (v2017.9) [34].

#### 2.8.3. Statistical Analyses

Variation in the composition of microbial communities between samples (beta diversity) was analysed using Redundancy Analysis (RDA) and Permutational Analysis of Variance (PERMANOVA) in R (v1.4.1717) [35] using the vegan package. Stacked community composition bar charts were created using a custom R script and heatmaps were generated using the heatmap function in R. Tukey’s Honest Significance Difference (HSD) was performed on ANOVA models to determine significant differences between sample cell counts, OTU abundance by treatment, and Shannon diversity indices.

## 3. Results

### 3.1. Absolute Abundances of Captured Cells

**Figure 1:**
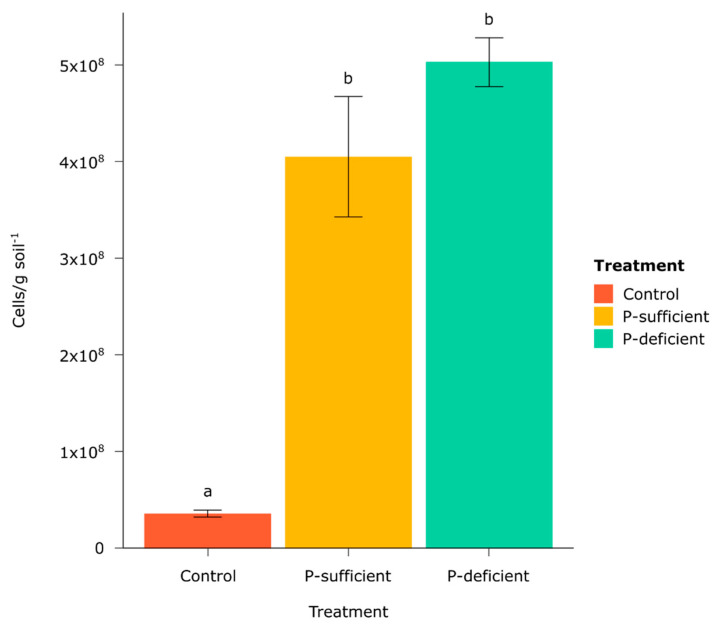
Mean number of cells captured (per gram of soil) in the chemotaxis assays by treatment after 30 min incubation. Different letters above bars represent statistically significant differences as determined by Tukey’s HSD (*p* < 0.05). Error bars represent the standard error of the mean.

Microbial cell counts from chemotaxis assays in all P-sufficient and P-deficient exudate replicates were significantly higher than in the control treatment (Figure 1). No significant difference was observed in cell counts between exudate-baited treatments (Figure 1).

### 3.2. Identities of Captured Cells

**Figure 2:**
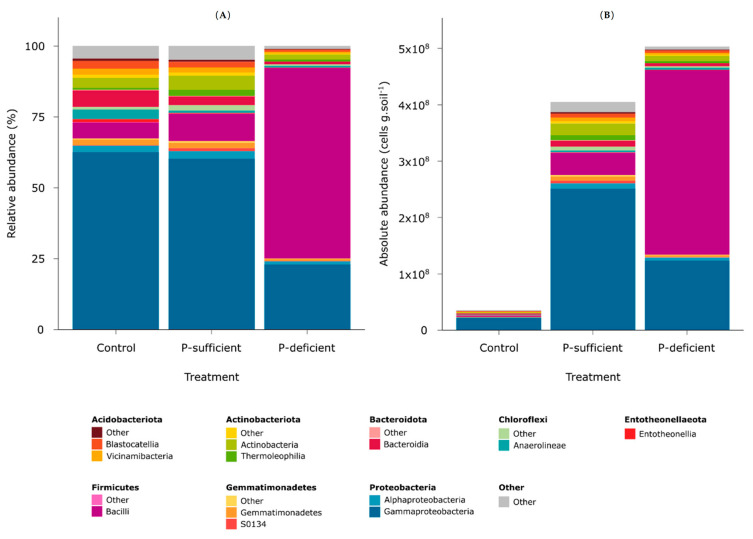
(**A**) Relative and (**B**) absolute (cell count-corrected relatives) abundances of bacterial communities captured in control, P-sufficient, and P-deficient treatments at the phylum and class levels.

Metabarcoding revealed that the captured microbial taxa spanned 29 bacterial phyla that varied in relative abundance according to the treatments (Figure 2). The control was dominated by *Proteobacteria* (~64.9%) followed by *Bacteroidota* (~6%) and *Firmicutes* (~5.8%). Exudates from P-sufficient plants attracted mostly *Proteobacteria* (~62.9%), *Firmicutes* (~9.6%), and *Actinobacteriota* (~8.2%). In contrast, exudates from P-deficient plants predominantly attracted *Firmicutes* (~67.2%), whereas considerably fewer *Proteobacteria* (~24.2%) and *Actinobacteriota* (~2.8%) were attracted compared to the control and P-sufficient exudates (Figure 2).

**Figure 3:**
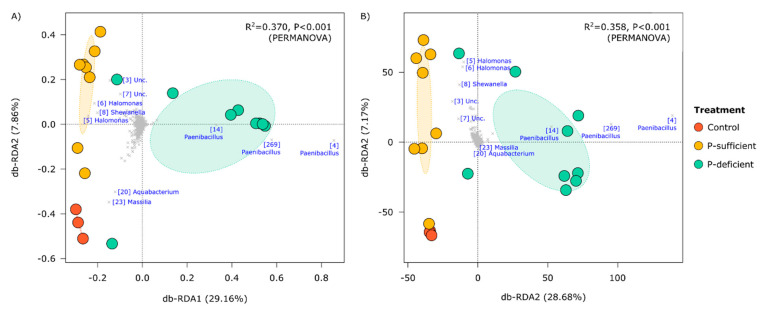
Distance-based redundancy analyses (db-RDA) summarising the compositional similarity of bacterial communities in control and exudate-baited traps as represented by Hellinger-transformed (**A**) relative, and (**B**) absolute abundances. In the top right of each panel are the results of PERMANOVA models in which treatment was a categorical predictor variable. Dominant operational taxonomic units (OTUs) identified at the genus level are shown in blue text with the OTU IDs in square brackets. These IDs are consistent between figures.

**Figure 4:**
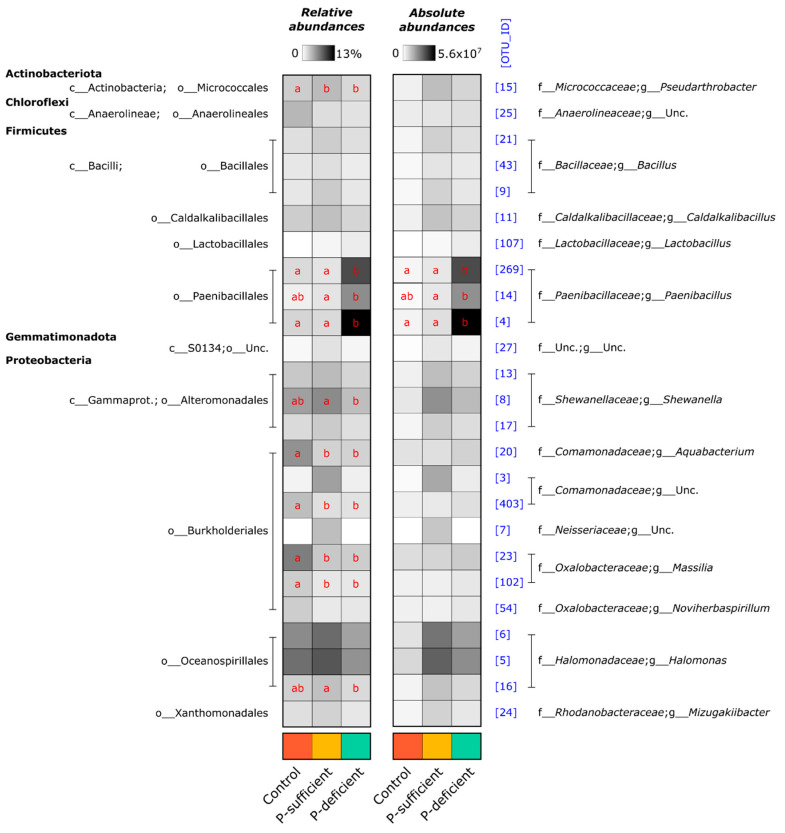
Heatmap summarising the relative and absolute-estimated abundances of bacterial OTUs present at ≥2% mean relative abundance in any treatment. Numbers in square brackets represent OTU IDs and are consistent with other figures. Red letters indicate the results of Tukey’s HSD post hoc tests for individual OTUs. Within OTUs, treatments sharing the same letter are not significantly different. Rows with no letters all shared the same letter.

At the OTU level, the composition of bacterial communities differed significantly between treatments (*p* < 0.001, PERMANOVA), with P-deficient exudates attracting significantly more *Paenibacillus* (OTUs 4, 269, and 14) populations relative to other treatments (Figure 3 and Figure 4). Bacterial communities attracted to P-sufficient exudates were associated with larger abundances of two *Halomonas* populations (OTUs 6 and 8), a representative of the *Shewanella* (OTU 8), and two unclassified taxa belonging to *Comamonadaceae* and *Neisseriaceae* families (OTUs 3 and 7) (Figure 3 and Figure 4).

The relative abundances of the most dominant OTUs captured in at least one of the chemotaxis assays are shown in Figure 4. Of the five phyla represented (*Actinobacteriota*, *Chloroflexi*, *Firmicutes*, *Gemmatimonadota,* and *Proteobacteria*), the *Firmicutes* (specifically, OTUs assigned to the genus *Paenibacillus*) were the most abundant group attracted to the P-deficient exudate treatment. In contrast, the *Proteobacteria* (specifically, members of the genera *Halomonas* and *Shewanella*) were the most abundant group attracted to the P-sufficient exudate treatment. The *Paenibacillus* genus differed between P-sufficient and P-deficient exudate treatments in both relative and absolute-estimated abundance (Figure 4). In addition, *Paenibacillus* OTUs represented 64.3% of the community elicited by P-deficient exudates, yet only 1.9% and 2.1% of communities were captured in P-sufficient and control treatments, respectively (Figure 4).

### 3.3. Alpha Diversity of Captured Cells

**Figure 5:**
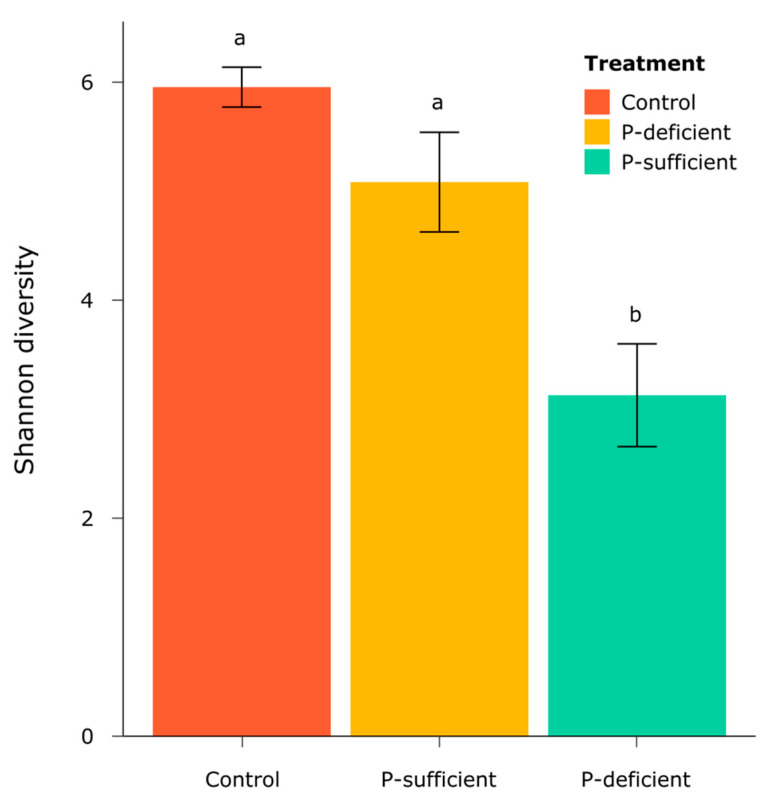
The alpha diversity of bacterial communities, as represented by Shannon’s Diversity Index, in each treatment. Error bars are standard errors, and the letters represent the results of Tukey’s HSD post hoc analysis, where treatments with the same letter are not significantly different (*p* < 0.05).

The alpha diversity of bacterial communities, as represented by Shannon’s Diversity Index, differed significantly between treatments (*F* = 7.76, *p* = 0.04, ANOVA), with P-deficient exudates attracting a significantly less diverse bacterial community than the control and P-sufficient treatments (Figure 5).

## 4. Discussion

The chemical dialogue between plants and microbes has co-evolved to mutually enhance the fitness of plants and microbes to prevailing abiotic and biotic conditions [17]. It is not only known that plants can alter the abundance and composition of their root exudates in response to stress, but that these changes can influence the microbial communities found at the root–soil interface [36]. Numerous studies highlight how root-associated microbes can enhance plant nutrient acquisition [18,19]. However, our understanding of how these microbes are drawn to root exudates relies heavily on culture-dependent methods [21,22,37,38,39]. These methods involve growing individual microbial species in standard culture media. As only a small proportion of the entire microbial community can be cultivated using these methods [40], this leaves us with a significant gap in understanding the collective chemotactic responses of soil microbes towards plant exudates. Here, we profiled the attraction of microorganisms to plant root exudates at the community level. Using a culture-independent bait-trap chemotaxis assay adapted from Dennis et al. [23] along with flow cytometry and 16S rRNA marker gene sequencing, we captured, enumerated, and characterised bacterial cells attracted to the exudates of P-sufficient and P-deficient plants and compared these to an exudate-free control solution with uniform chemical properties.

As P quickly becomes organically bound to soil, plants recover less than 20% of applied agricultural P [41,42]. Improving nutrient efficiency would contribute to higher yields and reduce financial and environmental costs associated with fertilisers. The attraction of bacterial populations with P-solubilising traits towards exudates from P-stressed plants could also provide a valuable mechanism for microbially mediated P solubilisation in agriculture.

Chemotaxis assays containing plant root exudates—from P-sufficient and P-deficient plants—recruited significantly more microbial cells than the exudate-free control. Many studies have reported that the increased microbial abundance and activity in the rhizosphere compared to the bulk soils is mainly due to the release of carbon-rich and energy-yielding compounds through rhizodeposition [43,44,45]. While P-deficient and P-sufficient exudates attracted similar numbers of bacteria, the composition of the communities that they attracted was significantly different. P-deficient root exudates predominantly attracted *Firmicutes* OTUs belonging to the genus *Paenibacillus*. In contrast, the control and P-sufficient treatments attracted mainly *Proteobacteria* OTUs assigned to the genera *Halomonas* and *Shewanella*. The relative abundance of *Paenibacillus* was over 30 times higher in the P-deficient treatment (~64.3%) compared to the P-sufficient treatment (~1.9%) and the control (~2.1%). P-deficient root exudates also attracted a less diverse community, suggesting higher specificity in the taxa recruited by exudates from P-stressed plants. In contrast, both P-sufficient and control treatments elicited positive responses from a wider range of taxa, indicating less microbial selectivity in their exudate profiles.

Importantly, members of the *Paenibacillus* are reported to be among the most powerful P solubilisers, facilitators of plant growth promotion, and effective agents in phytopathogen control [42,46,47,48,49,50]. *Paenibacillus macerans*, for instance, not only showed substantial P solubilisation but significantly increased plant P uptake when co-inoculated with arbuscular mycorrhizal fungi, supporting the notion that these counterparts exert a synergistic effect on plant health and nutrition [51].

Comparative genomic and functional analyses indicated that the majority of the 35 studied *Paenibacillus* strains possess genes responsible for the solubilisation of inorganic mineral phosphates through the production of gluconic acid [46,52]. A wide range of other plant growth-promoting traits have also been experimentally identified in *Paenibacillus* strains including nitrogen fixation as well as the production of organic acids, ACC deaminase, indole-3-acetic acid (IAA), and siderophores [42,47]. Among the detection of chitinolytic, antifungal, and P solubilisation traits in *Paenibacillus elgii*, Das et al. (2010) demonstrated increases of ~56%, 72%, and 92% in the shoot height, root length, and biomass of tobacco plants, respectively, when inoculated with the strain [49]. Additionally, they reported a 72.3% increase in germination rate for inoculated groundnut seeds. Recent reports have indicated that P-solubilising *P. polymyxa* could even withstand harsh environmental conditions [42]. For instance, the inoculation of *A. thaliana* with the P-solubilising *Paenibacillus yonginensis* DCY84^T^ promoted host tolerance to various abiotic stressors, including salinity, drought, and heavy metal contamination [53].

Abundant experimental evidence has demonstrated the antimicrobial properties of *Paenibacillus* strains, such as *Paenibacillus polymyxa*, inhibiting the growth of *Botryosphaeria dothidea*, *Botrytis cinerea*, *Fusarium fujikuroi*, *Fusarium oxysporum,* and *Pythium* plant pathogens [42,47,54]. *P. polymyxa* has also proven successful in suppressing *Phytophthora* and enhancing pathogenesis-related (PR) protein gene expression in pepper plants [50], revealing its versatile role in conferring plant protection and promoting plant growth. Evidently, *Paenibacillus* strains harbour valuable mechanisms for plant growth promotion and protection and demonstrate an important role in the plant–microbe chemical dialogue.

In a recent study employing exudate collection methods similar to those in this research, significant differences were observed in the root exudate patterns of Arabidopsis plants during their vegetative and bolting phases under varying phosphate fertilisation [55]. The study noted a substantial increase in the cumulative root secretion of malic acid in Arabidopsis plants during the bolting phase under low phosphate levels. Interestingly, another study has shown that malic acid can recruit the rhizobacterium *P. polymyxa* SQR-21 and increase its population on the root surface of watermelon [56].

The approach we propose facilitates future studies that link pure compounds or compound mixtures with the attraction of specific beneficial microbes. It can also accelerate targeted screening and isolation of these helpful chemotactic microbes. The application of identified compounds to stressed plants can act as prebiotics by selectively attracting key microbes to the plant. Similarly, applying inoculants formulated with these key microbes can increase the abundance of beneficial strains in the plant-associated microbial community. This approach holds significant potential for soil microbiome engineering at the community level, thereby promoting plant resilience to stress.

## 5. Conclusions

The results of this study, in conjunction with the novel culture-independent bait-trap assay, provide a foundation for further scientific exploration. Microbial chemotaxis is widespread among soil microbes and plays a pivotal role in mediating plant–microbe interactions. Characterising microbial attraction towards plant root exudates exposed the selective influence of plants on the composition and diversity of soil microbial communities under P limitation. The less diverse microbial community attracted to P-deficient plant root exudates, dominated by the *Paenibacillus* genus, highlights the specificity of plant–microbe chemical communication. Equipped with P solubilisation capabilities and other plant growth-promoting traits, these findings indicate the role of exudates in attracting populations with mechanisms to support plants under P-limited conditions. As microbial chemotaxis is ubiquitous in nature, this methodology could prove a powerful tool for the isolation and characterisation of microbes elicited by chemical cues and holds potential for microbiome engineering.

## Data Availability

All data are available on request.
